# Oral microbiome dysbiosis in acute ischemic stroke and transient ischemic attack patients

**DOI:** 10.1371/journal.pone.0333676

**Published:** 2025-10-07

**Authors:** Duangnapa Roongpiboonsopit, Sirikanya Wairit, Chichaya Nithisathienchai, Akkharin Pakdee, Thanya Cheibchalard, Jarun Sayasathid, Alisa Wilantho, Sissades Tongsima, Naraporn Somboonna

**Affiliations:** 1 Division of Neurology, Department of Medicine, Faculty of Medicine, Naresuan University, Phitsanulok, Thailand; 2 Stroke Unit, Naresuan University Hospital, Phitsanulok, Thailand; 3 Quality Assurance Department, Faculty of Medicine, Naresuan University Hospital, Phitsanulok, Thailand; 4 Department of Microbiology, Faculty of Science, Chulalongkorn University, Bangkok, Thailand; 5 Cardiac Center, Naresuan University Hospital, Naresuan University, Phitsanulok, Thailand; 6 National Biobank of Thailand, National Center for Genetic Engineering and Biotechnology (BIOTEC), National Science and Technology Development Agency, Pathum Thani, Thailand; 7 Multi-Omics for Functional Products in Food, Cosmetics and Animals Research Unit, Chulalongkorn University, Bangkok, Thailand; 8 Microbiome Research Unit for Probiotics in Food and Cosmetics, Chulalongkorn University, Bangkok, Thailand; Monash University Malaysia, MALAYSIA

## Abstract

Oral microbiome (bacterial community) may influence systemic inflammation and vascular health, which both are critical factors in a pathogenesis of ischemic stroke. This study aimed to evaluate differences in the saliva microbiome of acute ischemic stroke (AIS) and transient ischemic attack (TIA) patients compared with matched healthy controls, hypothesizing that AIS and TIA patients are associated with oral microbiome shift. A prospective case-control study was conducted in Naresuan University Hospital, Thailand, to compare the saliva microbiome of AIS and TIA stroke patients of Thai ethnic with matched healthy controls. Microbial profiles were analyzed by metagenomics combined 16S rRNA gene sequencing to assess microbial alpha diversity, taxonomic composition, beta diversity, and microbial functional pathways.Forty-one patients (31 AIS and 10 TIA) and 20 age- and sex-matched stroke-free healthy controls were included in this study. Baseline characteristics were comparable between groups, apart from higher rates of hypertension, diabetes, and smoking in the patient group. Patients exhibited significantly higher alpha-diversity genus richness by OTUs and Chao1 index than controls (p < 0.001), highlighting an altered microbial community structure. Phylum-level analysis revealed an increased abundance of Bacillota (p = 0.0285) in the patient group, with a statistically decreasing trend for Bacteroidota, Actinomycetota and Pseudomonadota (p < 0.05). At the genus level, *Streptococcus* was more significantly abundant in the patients (p = 0.0171), while *Prevotella* was reduced. The patient and control groups were statistically separated in beta-diversity analysis (PERMANOVA, p < 0.001), with species biomarker analysis by LEfSe (Linear discriminant analysis effect size) could suggest species markers for each group. Functional pathway analysis showed the patient group the significantly higher in functional categories of, for examples, xenobiotics biodegradation and metabolism, cardiovascular diseases, signal transduction, and membrane transport (Welch’s *t*-test, p < 0.05). In conclusion, *t*his study demonstrated the statistical alterations in the saliva microbiome of AIS and TIA patients, characterized by increased genus richness diversity and relatively distinct microbial shifts that may be associated with stroke-related inflammation. The findings suggest the saliva microbiome analysis as potential as a non-invasive biomarker for stroke risk and its role in stroke pathophysiology.

## Introduction

Acute ischemic stroke (AIS) is a leading cause of mortality and long-term disability globally, impacting substantially to healthcare burdens [1,2]. Although well-established risk factors, including hypertension, diabetes, smoking, and dyslipidemia, play significant roles in stroke pathogenesis, emerging researches suggest that the dysbiosis in microbiome (bacterial community) may also involve stroke susceptibility and outcomes [3–5].

The human microbiome has increasingly been implicated important in various systemic diseases, including cardiovascular and cerebrovascular conditions. While the gut microbiome has been widely studied in stroke patients [5] and revealed potential links with immune dysregulation and systemic inflammation, the role of the oral microbiome, specifically the saliva microbiome, remains relatively unexplored. The oral cavity, home to a diverse microbial community, has been reported to influence systemic health through inflammatory pathways and bacteremia, making it as potential contributor to vascular health and stroke risk [6]. Alterations in the oral microbiome, or oral dysbiosis, may contribute to systemic inflammation, endothelial dysfunction, and atherosclerosis, which these are key factors in stroke development [7]. Since saliva can be collected easily and non-invasively, and may accurately reflect oral microbial health [8], it serves as an ideal medium for investigating potential associations between the oral microbiome and stroke pathophysiology. Dysbiosis shifts in microbial diversity and composition in the saliva of AIS and transient ischemic attack (TIA) stroke patients may reveal insights into inflammatory mechanisms relevant to stroke. Both AIS and TIA involve a blockage of blood flow to the brain, causing temporary (for TIA) or permanent (AIS) brain damage, respectively, and that the oral salivary microbiota may be associated with AIS and TIA stroke risk [9–11].

This study thereby investigated the differences in the saliva (oral) microbiome of AIS and TIA patients compared to healthy controls, focusing on microbial diversity, taxonomic composition, and microbial functional pathways. We hypothesize that stroke patients exhibit a distinct microbial profile that may contribute to systemic inflammation and immune modulation associated with stroke pathogenesis. The findings from this research supported further understanding of the oral microbiome’s diversity and role in cerebrovascular diseases, and may support future development for oral microbial biomarker and/or supportive therapeutic strategies for stroke prevention and management.

## Materials and methods

### Standard protocol and ethical approvals

This study was approved by the institutional review board (IRB) of the Naresuan University Hospital (IRB No. P3-0020/2565), following the Declaration of Helsinki. Written informed consents were obtained from all participants before collecting all data.

### Study design and population

This prospective, case-control study was conducted at Naresuan University Hospital, Phitsanulok, Thailand, from August 2022 to October 2023. Sixty-one total subjects were included: 41 patients were consecutively recruited from the Acute Stroke Unit, while 20 controls were recruited from the Check-up Unit. Acute ischemic stroke (AIS) patients were defined as those with acute focal neurological deficits evidenced by brain infarction confirmed via computed tomography (CT) or magnetic resonance imaging (MRI). Transient ischemic attack (TIA) was defined acute focal neurological deficits lasting less than 24 hours without brain infarction on MRI scans. Inclusion criteria included: 1) age ≥ 45 years, 2) onset of stroke symptoms < 72 hours, 3) admission to the stroke unit, 4) available CT or MRI, and 5) written informed consent. Control participants were consecutive healthy individuals aged ≥ 45 years who attended annual check-ups and were matched to patients by age (± 5 years) and gender. Controls were recruited in a 2:1 ratio to stroke patients.

Exclusion criteria for both groups included: 1) altered consciousness or inability to provide consent, 2) history of stroke (controls only), 3) recent antibiotic use, 4) active infection, 5) temperature > 38°C, 6) white blood cell count > 15,000 cells/mm^3^, 7) cancer history, 8) abnormal kidney function (serum creatinine > 2 mg/dL), 9) autoimmune diseases, and 10) pregnancy or lactation. Demographic data and clinical data were collected from medical records and patient interviews. Data included gender, age, height, weight, history of hypertension, history of diabetes mellitus, history of dyslipidemia, smoking, history of previous stroke (for stroke patients). Laboratory investigation including fasting blood sugar (FBS), total cholesterol (TC), triglyceride (TG), low density lipoprotein (LDL), high density lipoprotein (HDL), blood urea nitrogen (BUN), creatinine (Cr), were measured. For stroke patients, etiology of stroke was classified according to the Trial of ORG 10172 in Acute Stroke Treatment (TOAST) criteria [12]. Stroke severity was assessed using the National Institute of Health Stroke Scale (NIHSS), and functional outcome was evaluated using the modified Ranking Scale (mRS) on admission and at discharge [13].

### Saliva sampling collections

Saliva samples were collected in the morning before breakfast from the control group and within 72 hours of stroke onset (before any meal) in the patient group. Participants were instructed to sit comfortably, rinse their mouths with water, and spit 3 mL of saliva into a 50 mL sterile Falcon tube. Samples were then frozen at −20°C and transferred under continuous cold storage to the Microbiome Research Unit for Probiotics in Food and Cosmetics at Chulalongkorn University, Bangkok, Thailand.

### Microbial analysis by metagenomic extraction and 16S rRNA gene sequencing

Metagenomic DNA was extracted from 1 mL of each sample and centrifuged (5,000 × *g*) for 5 minutes [14]. The cell pellet was extracted with a DNeasy PowerSoil kit according to the manufacturer’s instructions (Qiagen, Maryland, USA). DNA quantity and quality in the extracted metagenomes were determined by nanodrop spectrophotometry (A260 and A260/A280) and agarose gel electrophoresis. The metagenomic DNA was stored at −20°C.

Metagenomic DNA was used as a template for 16S rRNA gene V3–V4 region library preparation. Universal prokaryotic primers 341F (5′-CCTACGGGNGGCWGCAG-3′) and 805R (5′-GACTACHVGGGTATCTAATCC-3′) were used [15,16]. In brief, the 341F–805R PCR mixture comprised 2 × SparQ HiFi PCR Master Mix (QuantaBio, Massachesetts, USA), 0.2 μM 341F primer, 0.2 μM 805R primer, and 30 ng of the template. The PCR conditions were as follows: 98°C for 2 minutes, followed by 30 cycles of 98°C for 20 seconds, 60°C 30 seconds, 72°C for 60 seconds, and 72 °C for 1 minute. Then, each 16S rRNA gene V3–V4 amplicons were purified using SparQ Puremag Beads (QuantaBio), and appended index and barcode sequences via 8–10 cycles of PCR using 0.5 μM of Nextera TX index primers with the same PCR conditions. The final products were purified and pooled for the 2 × 250 bp next generation sequencing performed according to the Illumina MiSeq protocols (Illumina, San Diego, CA, USA) [17] at the Omics Sciences and Bioinformatics Center, Chulalongkorn University (Bangkok, Thailand).

### Bioinformatic and statistical analyses

Bioinformatic analyses for bacterial microbiota diversity and potential metabolisms. Raw sequences were processed using Mothur version 1.46.1 according to the standard operating procedures for MiSeq [18,19]. Quality sequences were obtained by removing sequences with <100 base pairs or ≤ 8 ambiguous bases and chimera sequences, and were aligned to reference genomes in SILVA version 138.1 (updated taxonomic classification to SILVA version 138.2) and Greengenes database version 13.8 for removal of non-prokaryote sequences (mitochondria, chloroplast, eukaryote, and unclassified sequences). The quality sequences were phylotype-classified operational taxonomic units (OTUs) into phylum (abbreviated p_), class (c_), order (o_), family (f_), genus (g_) and species (s_) levels; and since the number of quality reads differed across samples, we normalized (rarefied) all samples to an equal sequencing depth (37,483 quality reads per sample) (S1 Fig). Noted that, due to limitations in the sequence length of the 16S rRNA gene V3–V4 region (~465 bp) and database resolution, some OTUs could not be confidently assigned a species (or genus) name; meanwhile these were still distinct OTUs classified at the species-level rank, although unnamed. The Good’s coverage index (estimated percent sequencing coverage to true biodiversity), alpha diversity (numbers of OTUs, Chao richness and Shannon diversity), beta diversity non-metric multidimensional scaling (NMDS) based on the Jclass dissimilarity coefficients, and linear discriminant analysis effect size (LEfSe) for species biomarkers, were computed using Mothur 1.39.5 [20,21]. Microbial metabolic functions were predicted from the microbiota profiles using Phylogenetic Investigation of Communities by Reconstruction of Unobserved States (PICRUSt) (p < 0.05) and categorized on the basis of Kyoto Encyclopedia of Genes and Genomes (KEGG) pathways [22,23].

Clinical data statistics were performed using Stata/MP 14.0 software. Continuous variables with normal distributions are reported as mean ± standard deviation and were compared using an independent samples *t*-test. Categorical variables are presented as frequencies (percentages) and were analyzed with the χ^2^ test or Fisher’s exact test, as appropriate. Non-normally distributed continuous variables are reported as median (interquartile range). For microbiota statistics, *t*-test with centre-log-ratio transformation, permutational multivariate analysis of variance (PERMANOVA), or Tukey’s multiple comparisons test were used, and a p-value < 0.05 was considered significant [16,21]. For microbial metabolic potentials, the Statistical Analysis of Metagenomic Profiles (STAMP) was used [23]. Graph pictures were prepared by GraphPad Prism 9.0 (San Diego, California, USA).

## Results

Sixty-one total participants were enrolled, including 41 patients (categorized into 31 stroke and 10 TIA), and 20 matched healthy control participants. Baseline characteristics in age, gender, or BMI were comparable between the patient and control groups, with no statistically significant difference (Table 1, p > 0.05). However, the patients exhibited significantly higher systolic and diastolic blood pressure, and the higher prevalence of hypertension, diabetes, smoking, and lower LDL. Small vessel disease was the most common stroke subtype in the patient group. The median stroke severity in the stroke group, measured by NIHSS was 4 (Table 1).

**Table 1:** Clinical characteristics of patients and controls.

Clinical characteristics	Patients (n = 41)	Controls (n = 20)	p-value
Age, average years (±SD)	65.31 (9.46)	62.85 (7.70)	0.316
Male (n%)	26 (63.41)	11 (55)	0.584
Body mass index, kg/m^2^ (±SD)	23.88 (5.16)	25.59 (4.21)	0.205
Systolic blood pressure, mmHg (±SD)	147.75 (23.78)	128.15(13.12)	0.001
Diastolic blood pressure, mmHg (±SD)	86.76 (14.23)	74.5 (9.99)	0.001
Hypertension (n%)	29 (70.73)	4 (20)	< 0.001
Dyslipidemia (n%)	29 (70.73)	3 (15)	< 0.001
Diabetes mellitus (n%)	12 (29.27)	1 (5)	0.044
Smoking (n%)	14 (34.15)	0 (0)	0.002
Fasting blood sugar (±SD)	116.76 (40.94)	100.7 (16.84)	0.098
BUN (±SD)	23.07 (29.49)	23.10 (29.36)	0.997
Cr (±SD)	0.97 (0.29)	0.93 (0.23)	0.595
Total cholesterol, mmol/L (±SD)	187.22 (47.68)	209.8 (32.23)	0.060
Triglyceride, mmol/L (±SD)	141.49 (75.47)	138.25 (61.01)	0.868
HDL cholesterol, mmol/L (±SD)*	61.63 (44.18)	53.37 (12.13)	0.428
LDL cholesterol, mmol/L (±SD)*	97.17(48.20)	128.37 (29.16)	0.011
Stroke subtype by TOAST (n%)			
Large vessel atherosclerosis	10 (24.39)	–	
Cardioembolic stroke	6 (14.63)	–	
Small vessel disease	10 (24.39)	–	
Stroke of other determined etiology	1 (2.44)	–	
Stroke of undertermined etiology (n%)	4 (9.76)	–	
Transient ischemic attack (n%)	10 (24.39)	–	
NIHSS (median, IQR)	4 (1,6)	–	
mRS at admission (median, IQR)	3 (2,4)	–	
mRS at discharge (median, IQR)	2 (1,3)	–	

Abbreviations: BUN, Blood urea nitrogen; Cr, Creatinine; TOAST, Trial of Org 10172 in Acute Stroke Treatment; NIHSS, National Institute of Health Stroke Scale; and mRS, modified Ranking Scale; and * represented missing data = 1.

### Microbiota sequencing result and the compositions of oral microbiota between patients and control groups

The number of quality reads were high (average 98,238 reads in patient group, and 70,849 reads in control group), and hence all sequencing samples showed Good’s coverage indices at genus OTUs above 99% and the relatively plateau rarefaction curves at phylum and genus levels (S1 Table and S1 Fig), indicating sufficient sequencing depth to true biodiversity for microbiome data analysis.

**Fig 1:**
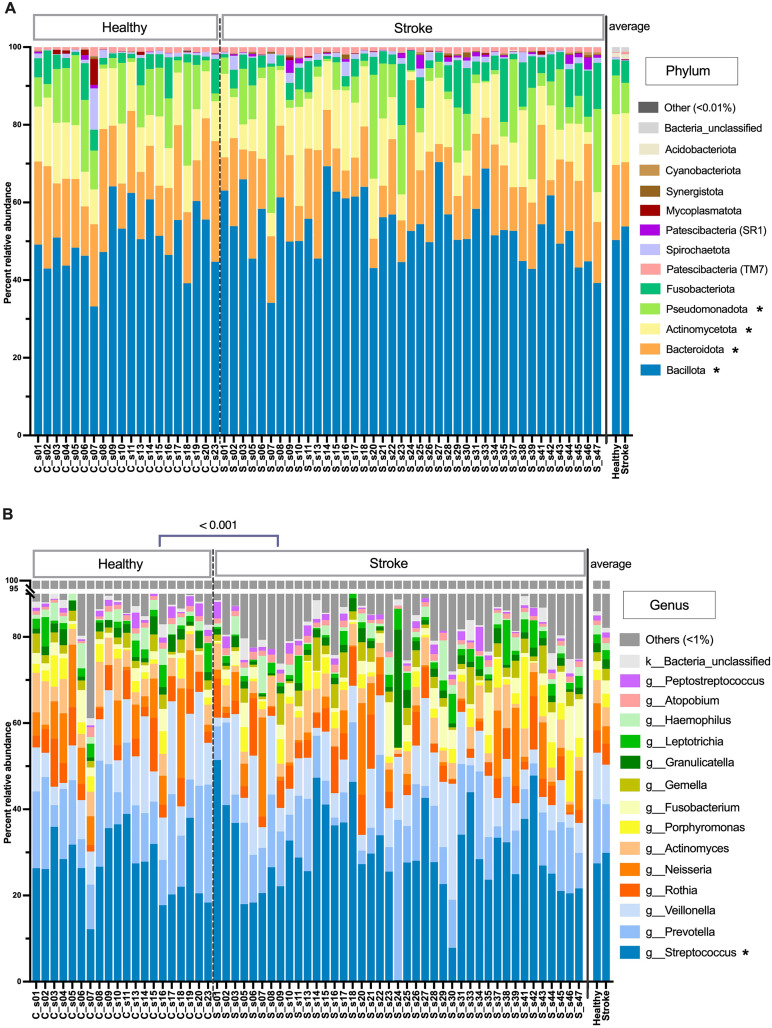
Microbiota compositions in percent relative abundance between healthy control and stroke patient individuals (the average microbiota between groups were displayed on the rightmost barcharts), at (A) phylum and (B) genus level OTUs. OTUs of less than 0.01% in phylum (or genus) were in Others. Symbol “*” after OTU name represented phylum (or genus) with the statistically different relative abundances between control and stroke groups (p < 0.05). In **(B)**, the OTU where Mothur could not identify the genus name was denoted by small letter to the deepest taxonomic names that could be identified (k_ abbreviates kingdom; and g_, genus).

As for the compositions, phylum Bacillota predominated in both groups with the statistically higher in stroke individuals (Fig 1A: average 50.31 ± 7.96% in control group, 53.79 ± 8.49% in stroke group) (*t*-test, p = 0.0285), followed by Bacteroidota(19.36 ± 6.23% control, 16.57% ± 7.44% stroke; p = 0.0006), Actinomycetota (13.04 ± 2.81% control, 12.62 ± 5.48% stroke; p = 0.0058), Pseudomonadota (9.98 ± 6.96% control, 7.83 ± 8.51% stroke; p = 0.0013), and Fusobacteria (4.13 ± 3.17% control, 5.73 ± 4.11% stroke), in orderly. While phylum Bacillota was significantly more abundant in stroke patients, Bacteroidota, Actinomycetota and Pseudomonadota showed a statistically decreasing trend; and Fusobacteria showed slightly more prevalent in stroke group (Fig 1A).

The genus level OTUs demonstrated the statistically significant difference in the overall microbiota compositions between the control and stroke groups (PERMANOVA, p < 0.001) (Fig 1B). *Prevotella* was lower (average 14.87 ± 6.58% in control group, 11.30 ± 6.63% in stroke group) and *Streptococcus* was statistically higher (27.46 ± 7.43% control, 29.89 ± 10.57% stroke) (*t*-*t*est, p = 0.0171) in stroke group. Overall, genera *Streptococcus*, *Prevotella* and *Veillonella* (10.81 ± 5.88% control, 9.16 ± 6.07% stroke) predominated, followed by *Rothia*, *Neisseria*, *Actinomyces*, *Porphyromonas* and *Fusobacterium*, respectively (Fig 1B).

**Fig 2:**
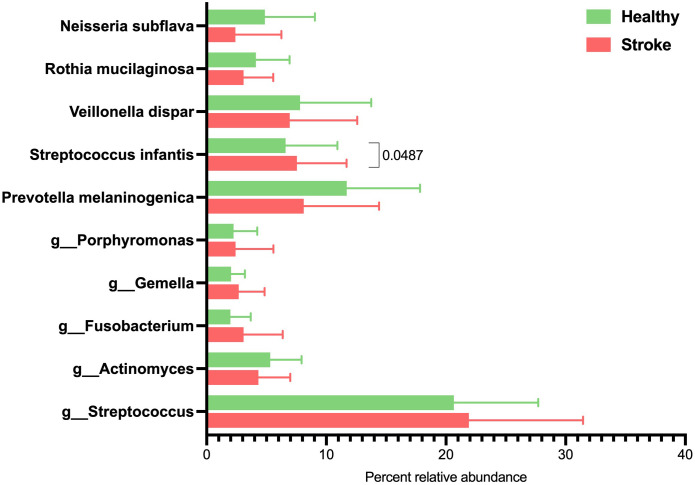
Average percent relative abundance of top 10 most abundance species of healthy control and stroke patient groups. The p-value was displayed only for the species with the statistically different relative abundances between groups (p < 0.05), and the OTUs where Mothur could not identify the species were denoted by small letter to the deepest taxonomic names that could be identified (g_ abbreviates genus).

Analyzing the top ten predominant species from the saliva samples belonged in genera *Streptococcus, Prevotella, Actinomyces, Fusobacterium, Veillonella, Gemella, Porphyromonas*, *Neisseria* and *Rothia* (Fig 2). A significant increase in the relative abundance of *Streptococcus infantis* (*t*-test, p = 0.0487) and a clear decrease in the relative abundance of *Prevotella melaninogenica* (p > 0.05), were observed in the stroke group.

### Alpha and beta diversity analyses

**Fig 3:**
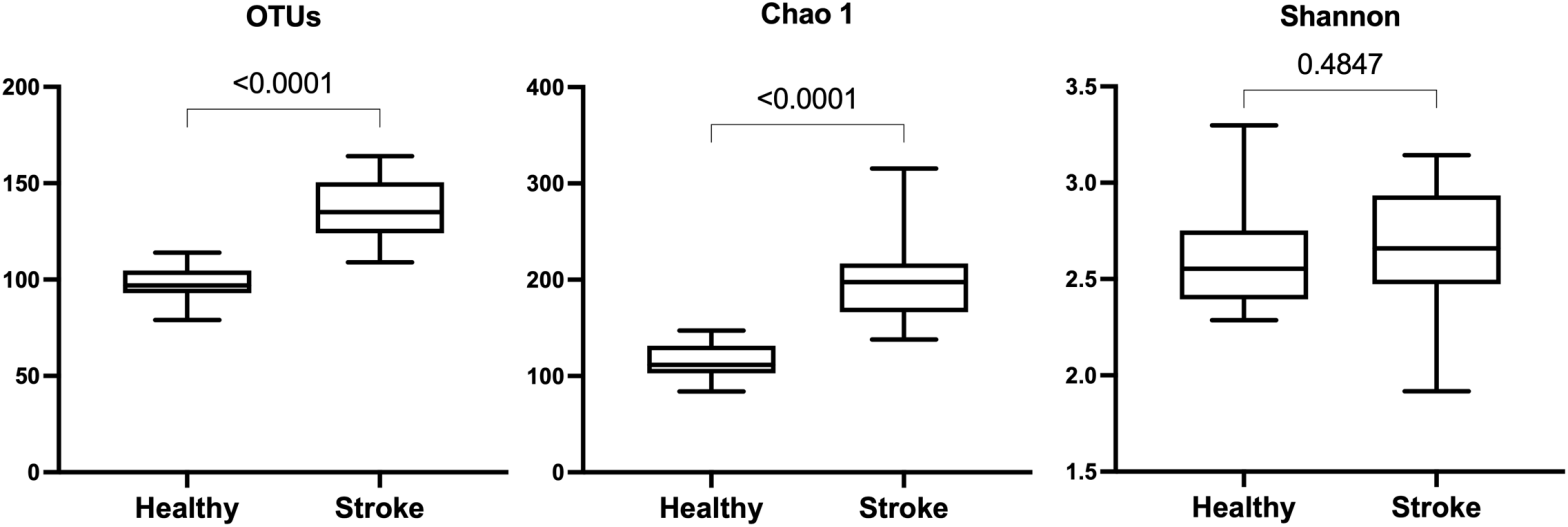
Alpha-diversity analysis at genus level including operational taxonomic unit (OTUs), Chao richness and Shannon diversity indices. Statistic comparisons were performed by *t*-test between patient and healthy control groups (p < 0.05).

In alpha diversity analysis to assess the diversity degree in individual samples, the Shannon diversity showed non-different diversity. Nonetheless, the number of OTUs and Chao1 genus richness were greater in the patients (p < 0.001) (Fig 3), highlighted the greater numbers of OTUs in stroke patients.

**Fig 4:**
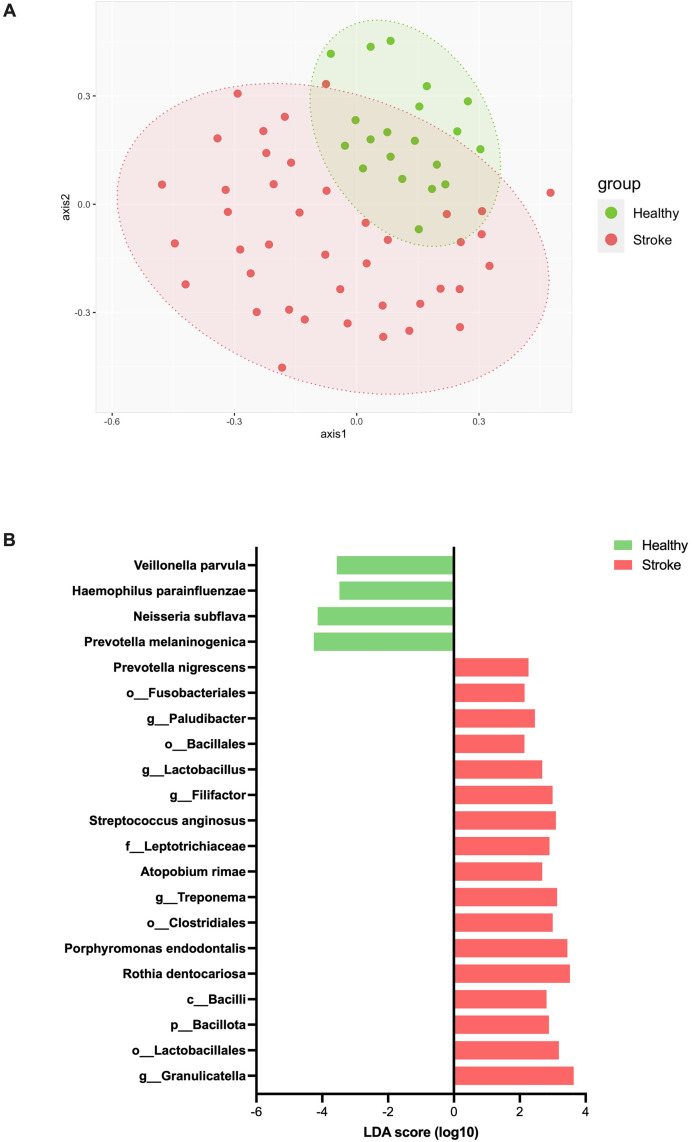
(A) Beta-diversity non-metric multidimensional scaling (NMDS) analysis at genus level, and (B) LEfSe for bacterial species biomarkers representing control vs. stroke groups.

Consistent with the statistic microbiota comparison between groups in Fig 1B, the beta diversity non-metric multidimensional scaling (NMDS) revealed relatively separate clusters for the control vs. the stroke groups (PERMANOVA, p < 0.001) (Fig 4A). The saliva microbiota structures in control individuals exhibited intra-group similarity, while wider distributions in stroke microbiota individuals might reflects their OTUs and Chao1 richness (Fig 4A).

### Oral bacterial species biomarkers for the control vs. stroke groups

Oral bacterial species OTU biomarkers determined by the linear discriminant analysis effect size (LEfSe) for the control vs. stroke groups, suggested *Prevotella melaninogenica, Neisseria subflava, Veillonella parvula* and *Haemophilus parainfluenzae* were enriched in the control group. Conversely, the stroke group was enriched in unidentified species in genus *Granulicatella, Porphyromonas endodontalis, Rothia dentocariosa, Streptococcus anginosus, Prevotella nigrescens* and *Atopobium rimae*, for examples (Fig 4B).

### Microbial metabolic potentials for the control vs. stroke groups

**Fig 5:**
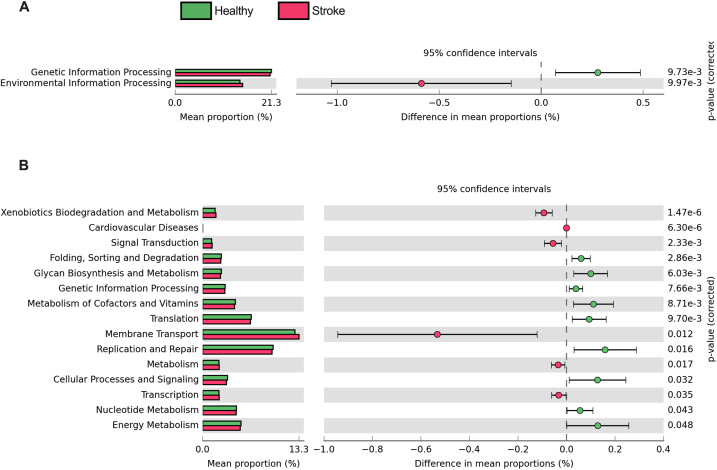
Microbial metabolic potentials categorized according to KEGG (A) level 1 and (B) level 2, representing control vs. stroke groups. Only the statistically different metabolic functions between groups were displayed (Welch’s *t*-test with Benjamini-Hochberg False Discovery Rate correction, p* *< 0.05).

Several differences in the microbial related metabolic processes were estimated between the control and stroke individuals, including genetic information processing and environmental information processing (Welch’s *t*-test, p < 0.05) (Fig 5A). For a subcategory (Fig 5B, KEGG level 2), the healthy control generally showed greater microbial metabolic potentials in most functions, for instances, replication and repair, energy metabolism, and cellular processes and signaling. In contrast, the stroke patients showed the greater in membrane transport, xenobiotics biodegradation and metabolism, cardiovascular diseases, and signal transduction (Fig 5B). Many of the microbial metabolic functions were consistent with the clinical characteristics of stroke (i.e., cardiovascular diseases, xenobiotics biodegradation and metabolism) vs. healthy (i.e., replication and repair).

## Discussion

Meanwhile some overlap similarities, this study highlighted differences in the oral (saliva) microbiome in AIS or TIA stroke patients compared with healthy controls, along their differential clinical characteristics. The data help provided a novel finding association between oral microbiome dysbiosis and stroke etiology.

The stroke individuals showed relatively significant greater number of OTUs and Chao1 richness, along the statistical distinct microbial compositions and beta diversity clustering, from the healthy controls, all indicating an oral saliva microbiome dysbiosis in the stroke compared with the healthy. The oral cavity of stroke patients may harbor a more diverse bacterial colonization, including potential pathogens and opportunistic pathogens, which could contribute to the acute post-stroke inflammatory environment and potentially influence systemic effects in AIS and TIA [6]. It is now well recognized that microbiome dysbiosis is associated with both disease and health [24,25]. Saliva microbiome diversity appears to vary across different stages of stroke. Preclinical studies have demonstrated an inverse association between microbial richness and stroke risk [26], whereas elevated diversity has been reported during the hyperacute phase (within 24 hours of stroke onset) [27] and the early subacute phase (within 2 weeks post-stroke) [28]. Our findings of increased diversity within 72 hours post-stroke further support the notion that microbial diversity dynamically shifts with disease progression and inflammatory responses.

At the phylum level, our study demonstrated an increase in Bacillota, particularly *Streptococcus*, and a decrease in Bacteroidota, Actinomycetota, Pseudomonadota, and *Prevotella* in stroke patients, consistent with findings from previous studies [27,28]. Elevated levels of *Streptococcus* have been associated with systemic inflammation and atherosclerosis, with *Streptococcus sanguinis* linked to aortic inflammation [29] and *Streptococcus viridans* implicated in the formation of atherosclerotic plaques [30]. Additionally, *Streptococcus mutans* carrying the *cnm* gene has been associated with cerebral microbleeds, suggesting a role in localized inflammation [31]. However, our results differ from those reported in cases of cryptogenic stroke, where *Prevotella* was found to be more abundant [32]. This discrepancy may reflect differences in stroke etiology, as cryptogenic stroke is often associated with non-atherosclerotic mechanisms and lower levels of systemic inflammation; and additionally, the difference in *Prevotella* is non-statistically significant in this finding.

Microbial-related functional pathway analysis predicted significant enrichment of pathways such as environmental information processing, cardiovascular diseases, signal transduction, membrane transport, and xenobiotics biodegradation and metabolism in the saliva microbiome of stroke patients. These shifts may reflect microbial adaptation to the pro-inflammatory and oxidative stress conditions present in AIS and TIA. In contrast, healthy controls exhibited higher predicted microbial functional potentials related to genetic information processing, energy metabolism, cellular processes and signaling, as well as replication and repair. These functions have been linked to microbial community stability and homeostatic support of host health in non-disease states [9,25]. It is important to note that such microbial functions were inferred using PICRUSt-based predictions from the 16S rRNA gene sequences, which estimated gene content and pathway potentials based on taxonomic profiles and reference genome databases [22,23]. Therefore, while these inferences are widely used, they represent approximations rather than direct functional measurements. The observed enrichment of predicted pathways involving xenobiotics metabolism and cardiovascular diseases in stroke patients also align with clinical features of stroke and systemic inflammation [33–35]. Meanwhile, the reduced potentials for replication and repair and energy metabolism in the stroke-associated microbiome may suggest signs of microbial community stress or dysfunction under pathological conditions [4,36]. Collectively, the results highlight the potential role of microbial dysbiosis contributes to disease progression by promoting inflammation and activating stress-response pathways and/or responses post-strokes.

Furthermore, the distinct microbial profiles in stroke patients suggest that saliva microbiome analysis could serve as a non-invasive screening for stroke risk stratification. Additionally, targeting the oral microbiome through interventions such as oral hygiene practices, dietary modifications, or probiotic therapies might help mitigate systemic inflammation and potentially reduce stroke risks [9–11].

Although direct causal links between the oral microbiome dysbiosis and stroke pathophysiology remain to be fully elucidated, this study findings parallel with patterns observed in other inflammatory-driven diseases. This study still posed limitations, including small sample size, and potential confounding factors such as diet, oral hygiene practices and periodontal status, which could influence microbiota compositions, and 1 sample (S_s15) was later diagnosed confounded by migraine with aura. While a thirteen-year longitudinal study in Thailand did not report a significant association between periodontitis and stroke incidence [37,38], oral health represents one key and may influence systemic inflammation. Future research should prospectively include dental and periodontal assessments to recruit this potential variable factor from the microbiota anaylsis. Additionally, the use of 16S rRNA gene sequencing, while valuable for taxonomic profiling, limits the full resolution of functional characterization and does not capture viral or fungal components of the microbiome.

Periodontitis is an inflammatory condition of the oral cavity that has been consistently associated with cardiovascular risk [6,37,38]. Oral pathogens such as *Porphyromonas*, *Prevotella*, *Fusobacterium*, and *Streptococcus* species can translocate into the bloodstream, and contributed to endothelial dysfunction, atherogenesis, and systemic immune activation [7,33]. In particular, *Porphyromonas endodontalis* and *Prevotella nigrescens*, which were enriched in our stroke cohort, are well-recognized periodontal pathogens that can stimulate pro-inflammatory cytokine production and have been implicated in both periodontal tissue destruction and vascular inflammation. Similarly, *Streptococcus anginosus* has been linked to oral infections and bacteremia, with potential cardiovascular involvement. Although we did not directly assess periodontal status in our participants, the overlap between stroke-associated oral taxa and known periodontal pathogens suggests a possible mechanistic bridge between periodontitis, oral microbiome dysbiosis, and stroke pathophysiology. Future studies should include alongside the dental and periodontal assessments to clarify this link and to evaluate whether periodontal management could mitigate stroke risk.

## Conclusion

This study demonstrated distinct alterations in the saliva microbiome profiles of patients with AIS and TIA compared to healthy controls. Stroke patients exhibited greater microbial richness, significant microbiota shifts, and enrichment of microbial pathways associated with inflammation, cardiovascular diseases, and environmental stress responses. These findings suggest that oral microbiome dysbiosis may contribute to systemic inflammatory environments and potentially influence stroke pathophysiology. Although causal relationships remain to be established, the observed associations highlight the potential of the oral microbiome both as a non-invasive biomarker for stroke risk assessment and as a novel target for supportive intervention and preventive care. Future large-scale, multi-center, and longitudinal studies incorporating multi-omics and functional analyses are warranted to further elucidate the role of the oral microbiome in stroke development and progression.

## Supporting information

S1 Fig Rarefaction curves of quality reads per sample at (A) phylum and (B) genus level operational taxonomic units (OTUs), in patient and control samples. Noted that since the number of quality reads differed across samples, we normalized all samples to an equal sequencing depth (37,483 quality reads per sample).
(TIFF)

S1 Table Saliva sample IDs, group, number of quality reads, Good’s coverage indices at genus OTUs, and subject information. (DOCX)
