# Supplementary figures and images for: Oral microbiome dysbiosis in acute ischemic stroke and transient ischemic attack patients
Source: PLoS One. 2025 Oct 7;20(10):e0333676. doi: 10.1371/journal.pone.0333676 (PMC12503293; doi:10.1371/journal.pone.0333676)

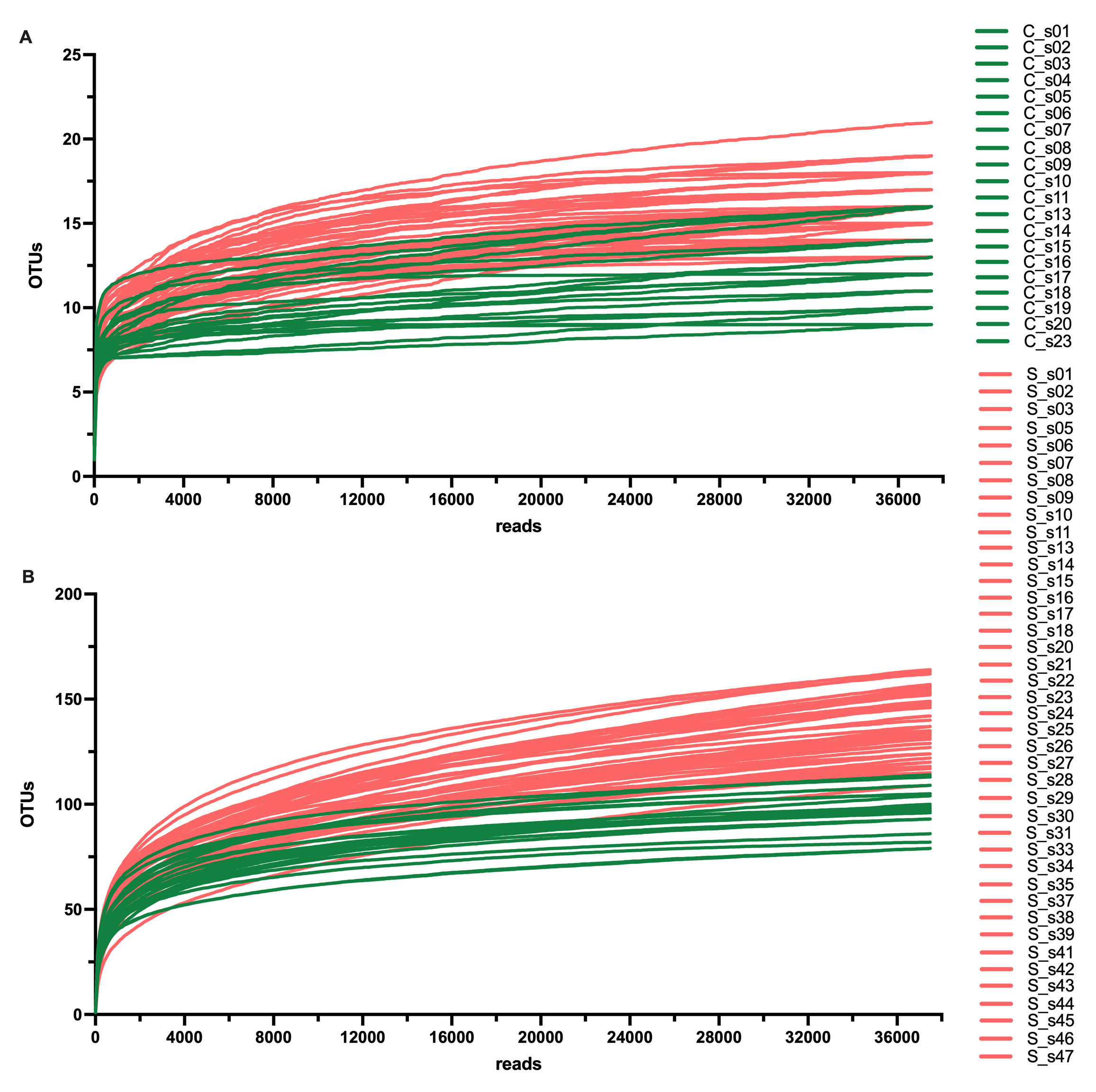

Supplement: S1 Fig — Noted that since the number of quality reads differed across samples, we normalized all samples to an equal sequencing depth (37,483 quality reads per sample). (TIFF) [file pone.0333676.s001.tiff]
