# Supplementary material for: Oral microbiome dysbiosis in acute ischemic stroke and transient ischemic attack patients
Source: PLoS One. 2025 Oct 7;20(10):e0333676. doi: 10.1371/journal.pone.0333676 (PMC12503293; doi:10.1371/journal.pone.0333676)
Supplement: S1 Table — (DOCX) [file pone.0333676.s002.docx]

| Sample | Group | Quality reads | Good’s coverage (%) | Sex | Age (years) |
| --- | --- | --- | --- | --- | --- |
| C_s01 | control | 79,415 | 99.95 | Female | 68 |
| C_s02 | control | 76,040 | 99.94 | Female | 67 |
| C_s03 | control | 74,692 | 99.93 | Male | 63 |
| C_s04 | control | 100,408 | 99.96 | Male | 64 |
| C_s05 | control | 67,776 | 99.97 | Female | 70 |
| C_s06 | control | 51,926 | 99.97 | Female | 47 |
| C_s07 | control | 49,072 | 99.95 | Male | 62 |
| C_s08 | control | 50,889 | 99.96 | Male | 60 |
| C_s09 | control | 37,483 | 99.96 | Female | 62 |
| C_s10 | control | 60,687 | 99.95 | Male | 56 |
| C_s11 | control | 89,012 | 99.98 | Male | 58 |
| C_s13 | control | 67,209 | 99.97 | Male | 66 |
| C_s14 | control | 65,116 | 99.97 | Male | 74 |
| C_s15 | control | 77,389 | 99.97 | Male | 43 |
| C_s16 | control | 69,582 | 99.98 | Female | 69 |
| C_s17 | control | 82,038 | 99.96 | Male | 68 |
| C_s18 | control | 58,209 | 99.96 | Female | 63 |
| C_s19 | control | 93,800 | 99.96 | Female | 72 |
| C_s20 | control | 93,618 | 99.95 | Female | 59 |
| C_s23 | control | 72,615 | 99.94 | Male | 66 |
| S_s01 | stroke(AIS) | 93,778 | 99.87 | Male | 56 |
| S_s02 | stroke(TIA) | 99,154 | 99.91 | Female | 74 |
| S_s03 | stroke(AIS) | 107,274 | 99.91 | Female | 69 |
| S_s05 | stroke(AIS) | 101,883 | 99.88 | Female | 67 |
| S_s06 | stroke(AIS) | 97,484 | 99.86 | Female | 72 |
| S_s07 | stroke(AIS) | 106,514 | 99.90 | Male | 67 |
| S_s08 | stroke(AIS) | 105,660 | 99.87 | Female | 88 |
| S_s09 | stroke(AIS) | 111,100 | 99.89 | Male | 64 |
| S_s10 | stroke(TIA) | 91,370 | 99.89 | Male | 63 |
| S_s11 | stroke(AIS) | 58,486 | 99.91 | Male | 75 |
| S_s13 | stroke(TIA) | 114,379 | 99.90 | Male | 71 |
| S_s14 | stroke(AIS) | 97,366 | 99.85 | Female | 58 |
| S_s15 | stroke(TIA) | 104,802 | 99.86 | Female | 49 |
| S_s16 | stroke(TIA) | 93,425 | 99.90 | Male | 62 |
| S_s17 | stroke(AIS) | 100,800 | 99.90 | Male | 74 |
| S_s18 | stroke(TIA) | 88,110 | 99.89 | Male | 57 |
| S_s20 | stroke(AIS) | 101,992 | 99.91 | Female | 77 |
| S_s21 | stroke(AIS) | 107,260 | 99.93 | Female | 68 |
| S_s22 | stroke(AIS) | 106,448 | 99.92 | Male | 75 |
| S_s23 | stroke(AIS) | 119,063 | 99.92 | Female | 70 |
| S_s24 | stroke(AIS) | 83,554 | 99.91 | Female | 55 |
| S_s25 | stroke(AIS) | 107,794 | 99.88 | Male | 63 |
| S_s26 | stroke(AIS) | 90,077 | 99.90 | Female | 70 |
| S_s27 | stroke(AIS) | 90,512 | 99.90 | Female | 71 |
| S_s28 | stroke(AIS) | 98,201 | 99.89 | Male | 48 |
| S_s29 | stroke(AIS) | 94,229 | 99.89 | Male | 74 |
| S_s30 | stroke(TIA) | 98,697 | 99.89 | Male | 77 |
| S_s31 | stroke(AIS) | 99,424 | 99.89 | Male | 58 |
| S_s33 | stroke(AIS) | 89,847 | 99.90 | Male | 63 |
| S_s34 | stroke(AIS) | 92,572 | 99.91 | Male | 59 |
| S_s35 | stroke(AIS) | 101,556 | 99.90 | Female | 59 |
| S_s37 | stroke(AIS) | 104,376 | 99.93 | Male | 61 |
| S_s38 | stroke(TIA) | 104,453 | 99.92 | Male | 55 |
| S_s39 | stroke(AIS) | 106,691 | 99.91 | Male | 72 |
| S_s41 | stroke(TIA) | 119,721 | 99.89 | Male | 46 |
| S_s42 | stroke(AIS) | 98,493 | 99.91 | Male | 71 |
| S_s43 | stroke(TIA) | 98,955 | 99.93 | Male | 74 |
| S_s44 | stroke(AIS) | 88,773 | 99.94 | Female | 51 |
| S_s45 | stroke(AIS) | 93,728 | 99.95 | Male | 60 |
| S_s46 | stroke(AIS) | 85,115 | 99.93 | Male | 79 |
| S_s47 | stroke(AIS) | 74,649 | 99.92 | Male | 56 |
